# Automation and validation of an NMR spectroscopy-based drug discovery assay

**DOI:** 10.1016/j.isci.2026.116555

**Published:** 2026-07-10

**Authors:** Thomas L. Allam, Matthew Rice, Jose Ortega-Roldan, Jens Unger, Perry Hailey, Dominick Balderston, Kira Hilton, Olivia Keers, Pinky Naicker, Precious Popoola, Mackenzie Stevens, Lisa-Jane White, Burkhard Schäfer, Jennifer R. Hiscock, Samantha Pearman-Kanza, Jeremy Frey

**Affiliations:** 1 University of Southampton, School of Chemistry and Chemical Engineering, Southampton SO17 1BJ, UK; 2 University of Kent, School of Natural Sciences, Canterbury CT2 7NZ, UK; 3 Splashlake, Robert-Bosch-Straße 7, 64293 Darmstadt, Germany

**Keywords:** high-throughput, drug development, automation, digitisation, searchable data, drug discovery, pharmaceutical development, NMR spectroscopy, supramolecular chemistry, computationally accessible data

## Abstract

The trillion-dollar drug development industry depends on high-quality data to guide decision making processes. Many drug candidates work by targeting specific biological sites within disease-causing cells, often entering via passive diffusion through the cell membrane(s). We have previously developed a solution-state ^1^H NMR spectroscopy assay that calculates comparative membrane adhesion and permeation factors for a drug candidate, aiding the design of effective, selective drugs that demonstrate minimal off-target (side) effects. However, the uptake of this assay into standardized drug development processes is limited because of the lengthy specialist manual workflows required to process the raw spectral data. Herein, we automate and validate the analysis of this assay, outputting high-quality data to remove this limitation, improving data accessibility, increasing assay throughput, experimental reproducibility, and interoperability with other workflows.

## Highlights

• NMR assay to understand the permeation of molecules, summarized as numerical factors
• Throughput limited by manual workflow to calculate the factor from the spectra analysis
• Validated automated workflow is within expected error, saving 223 s per experiment
• Automated workflow generates high-quality interactive XML summaries for analysis

## Graphical abstract

**Figure:**
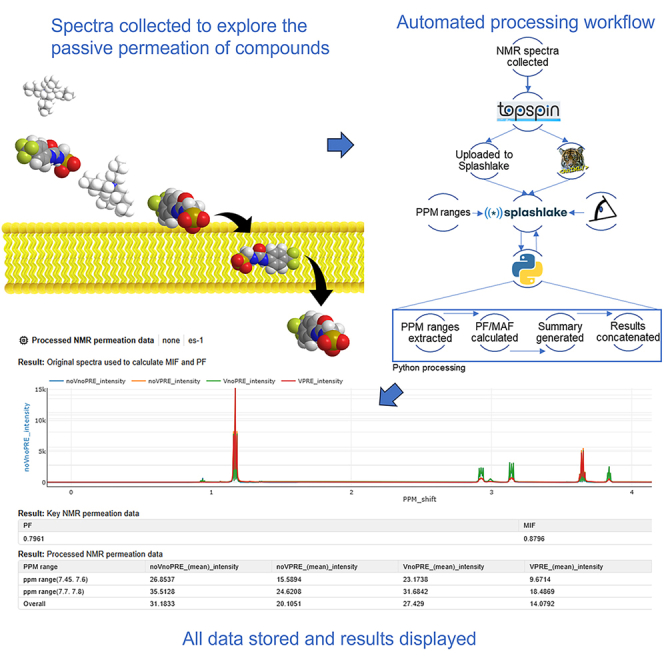

## Teaser

Biotechnology; Medical biotechnology; Biocomputational method; Pharmacoinformatics

## Introduction

Antimicrobial resistance (AMR) and cancer are two of the greatest health risks facing humanity today. Global AMR-related deaths were estimated at 4.71 million in 2019,^1^ which is predicted to rise to 10 million per year by 2050,^2^ due to several factors, which include but are not limited to ease of availability of antibiotics, excessive use in food-producing animals, and lack of resistance development surveillance.^3^ In comparison, 9.7 million deaths were directly attributed to cancer in 2022,^4^ which is predicted to grow to 18.5 million by 2050.^5^ This predicted rise in deaths is attributed to factors including the aging global population,^6^ and the generation of resistance to currently marketed anticancer agents.^7^ Therefore, there is an ever-present and growing need for the development of antibiotics and anticancer agents. However, the development of therapeutic agents is expensive, with the development of an antibiotic reportedly costing $1.2 billion.^8^ These are costs that are difficult to absorb within this area of the pharmaceutical sector, despite recent initiatives, due to diminished financial returns resulting from limited use of a safeguarded drug of last resort.^9^^,^^10^^,^^11^^,^^12^^,^^13^ In comparison, anticancer drug development costs can be more easily absorbed by this area of the pharmaceutical sector.^14^

However, the development of drugs in this area is limited by the undesirable side effects created when an anticancer drug elicits a biological effect on a normal non-disease cell. Therefore, ensuring anticancer drug cellular selectivity is incredibly important.^15^ The majority of drugs developed to treat both AMR and cancer elicit a therapeutic effect either through adhering/disrupting with the cell surface membrane(s), or by interacting with a biological target inside the disease cell, meaning that the drug molecule must gain cell entry by passing through the cell membrane(s), a process which is often facilitated via passive diffusion through the phospholipid bilayer. Interestingly, although the composition of the phospholipid bilayer is known to be very different between prokaryotic (e.g., bacterial) and eukaryotic (e.g., fungal or human) cells, a change in the phospholipid composition of mammalian cells is known to be one of the first physiological changes to be observed when a cell becomes cancerous.^16^

**Figure 1:**
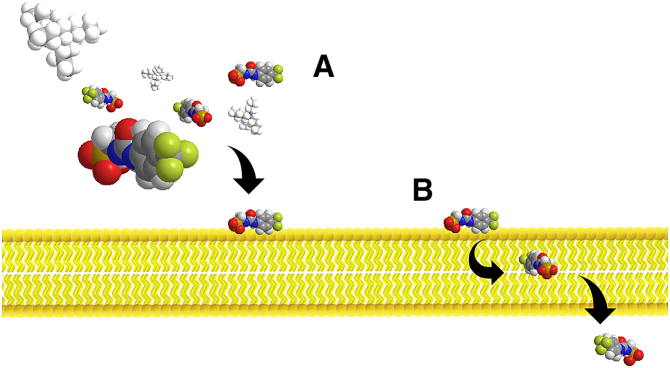
A cartoon illustrating (A) a drug candidate adhering to a phospholipid membrane (B) and subsequently permeating through the phospholipid membrane of a target disease cell.

To decrease the costs associated with drug development, we previously developed an accessible solution-state ^1^H NMR spectroscopy assay to remove these limitations. Through the calculation of parameters associated with the adhesion (membrane adhesion factor—MAF, Figure 1A) of drug candidates to, and permeation through (membrane permeation factor—PF, Figure 1B) cellular membranes; we can ensure a drug candidate reaches and successfully interacts with the desired biological target, while simultaneously supporting the development of drugs which display a greater degree of cellular selectivity toward a diseased cell over a non-diseased cell.^17^

The MAF and PF for a drug candidate are calculated from four different ^1^H NMR spectroscopy experiments, containing vesicles created from the phospholipids of target cells or commercial sources. Each of these different NMR experiments contains different combinations of vesicle (V), paramagnetic (PRE) NMR silencing agent, and drug/drug candidate.

The resultant ^1^H NMR spectra are first phased and baseline corrected automatically, with further manual phasing applied as necessary. The intensity from individual peaks on each spectrum is used to calculate the MAF and PF.^17^ Although this assay demonstrated the potential to transform anticancer and antimicrobial drug development processes, one limitation stopping the throughput is the time taken to run the analysis of the four NMR spectra, taking an average of 690 s.

In this paper, we address the limitations in analysis by automating the workflow to remove bottlenecks associated with both spectral processing and subsequent data analysis, thereby reducing the average analysis time by 223 s per set of four NMR spectra (termed the experiment), removing the data analysis time as a limitation of assay throughput and subsequent use. During this process, the original scientific findings were not challenged.^17^^,^^18^ To validate both the assay and the automated analysis, we reproduced the calculation of a subset of published MAF and PF values through reprocessing of the corresponding ^1^H NMR spectra. In addition, we implemented the necessary procedural and cultural changes, including the adoption of standardized naming conventions, to enable data centralization and metadata tagging that support automated analysis. This approach generates high-quality, searchable, and reproducible datasets in a manner that is generalizable to other workflows for audit and structure activity relationship studies.

### Methods

Outlines the tools and how we have automated the processing of ^1^H NMR 1D spectra to calculate MAF and PF, manual processing methods used, as well as how the two methods to validate the automated factor calculation (AFC), have worked as intended.

### Tools and software used

For the automation of the NMR spectroscopy permeation workflow, Splashlake Software^19^ was used as the data management system, while Python was used to process the data. For phasing, transformation, and baseline correction, a process for cleaning spectra before interpretation, the 1H NMR spectra, TopSpin software was used.^20^ Manually phased (MP) and automatically phased (AP) spectra were processed using the manual and AFC methods and compared to published results^17^^,^^18^ to ensure reproducibility and experimental validity.

### Automated data processing of NMR spectroscopy to calculate PF and MAF values

**Figure 2:**
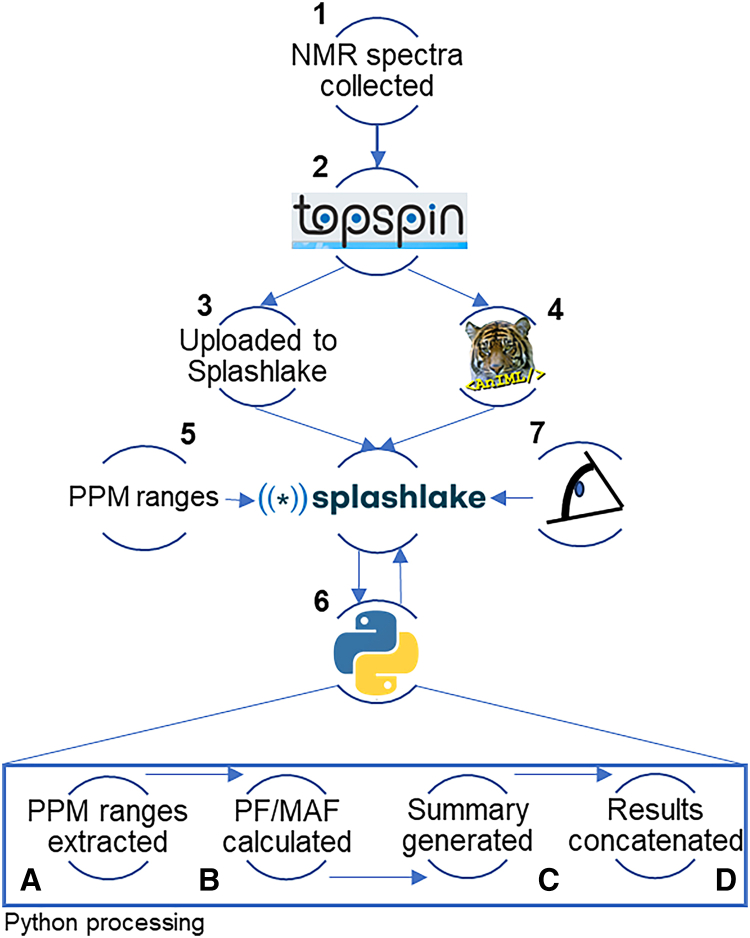
A flowchart to show an overview of the automated NMR permeation workflow A flowchart to show an overview of the automated NMR permeation workflow 1–7, with (A–D) the specific steps taken in the Python processing workflow.

**Figure 3:**
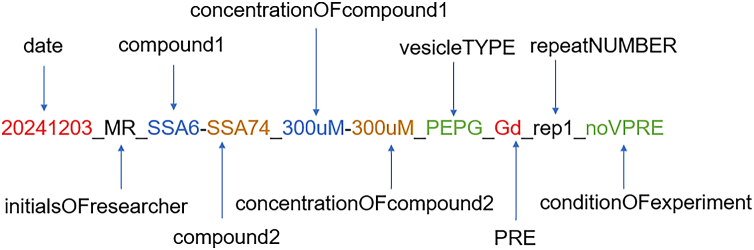
The file naming convention used in the NMR spectroscopy permeation workflow

Raw NMR spectroscopy data were obtained as .fid files for all four experimental conditions using a Bruker NMR spectroscopy 600 MHz machine fitted with a cryoprobe, Step 1 in Figure 2:

These data were opened in Topspin software (Figure 2, Step 2), where the spectra were also AP and baseline corrected (AP) using the following commands: efp, apk, and abs. Additional manual phasing and baseline correction (MP) was undertaken as necessary. The spectra folders were named using the naming convention shown in Figure 3, and saved to a monitored folder. The data, once copied to the monitored folder by the researcher, were automatically detected and subsequently uploaded to Splashlake, where processing was automatically triggered (Figure 2, Step 3). The files are tagged with metadata from the file name using regular expressions.

On upload, the. fid and ^1^H NMR 1D spectra files are programmatically transformed into Analytical Information Markup Language (AnIML)^21^ files for each experiment (Figure 3, Step 4). PPM ranges for each compound/Co-formulation were identified and added to the PPM ranges file by the researcher (Figure 2, Step 5). On generation, the AnIML files, containing the raw NMR spectra data for all four experimental conditions, and the PPM ranges file were sent, via an application programming interface call, directly to the Python processing scripts (Figure 2, Step 6). Summary files were then generated and uploaded back to the Splashlake data management system with the same naming conversions. All files are searchable by their metadata (Figure 2, Step 7).

**Figure 4:**
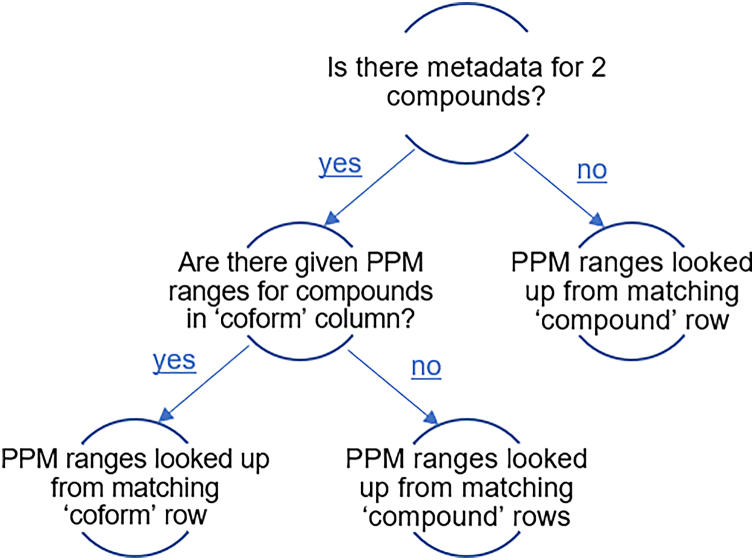
A flowchart detailing the method of automated PPM range identification.

Python is used for MAF and PF calculation, the PPM ranges are retrieved from the PPM ranges file, Step A (Step 3 and 5), using the metadata from the naming convention shown in Figure 4.

**Figure 5:**
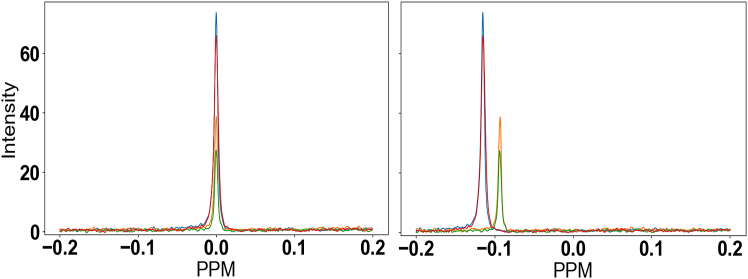
Overlaid ^1^H NMR spectra exemplifying spectral axis calibration. Right: ^1^H NMR spectra before standardization. Left: ^1^H NMR spectra referenced to 0 PPM, post standardization.

Step B, spectral data are extracted from the AnIML files and loaded into a DataFrame in Python, where the spectra axis was standardized around the peak of the ^1^H NMR spectroscopy standard (DSS in this study), as shown in Figure 5, between −0.2 and 0.2 PPM.

The DataFrame containing the calibrated spectra was searched over the defined PPM range. The maximum intensity calculated for each peak within the defined PPM range. The overall mean intensity for all PPM ranges with the experiment was then calculated. Equations 1 and 2 were used to calculate the MAF and PF using the intensity for each PPM range as well as the overall mean for all PPM ranges.

$$\text{MAF}=\frac{I_{\text{Vesicles}}}{I_{\text{no}\;\text{Vesicles}}}$$

Equation 1: An equation to show the calculation of MAF.

$$PF=\frac{{\left(\frac{I_{\text{PRE}}}{I_{\text{noPRE}}}\right)}_{\text{Vesicles}}}{{\left(\frac{I_{\text{PRE}}}{I_{\text{noPRE}}}\right)}_{\text{no}\;\text{Vesicles}}}$$

Equation 2: An equation to show the calculation of PF.

**Figure 6:**
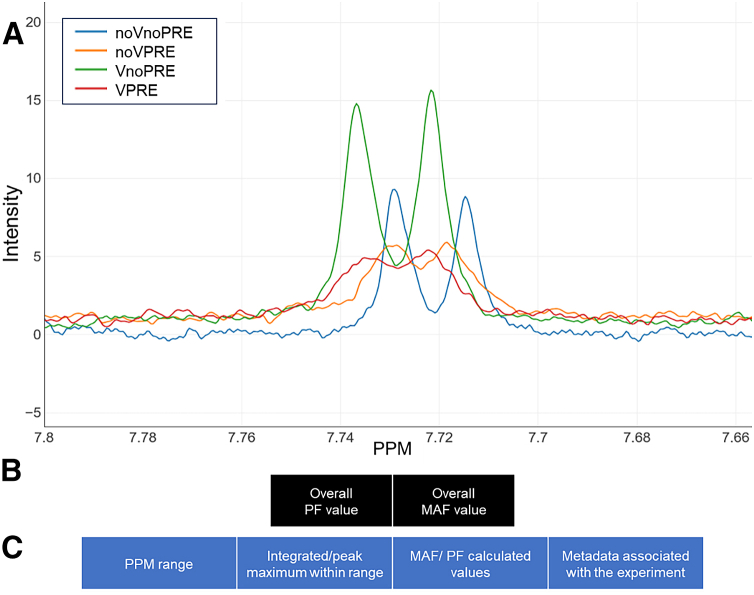
The output generated from the AnIML file as it is displayed in Splashlake. The file is split into three sections (A) The raw NMR spectra collected for the experiment displayed as an interactive graph; (B) the MAF and PF value; (C) summary of all metadata.

All metadata related to the experiment, alongside the calculated MAF and PF values, were added to the results DataFrame for each peak range. These data are saved as both CSV and AnIML files, which are uploaded to the Splashlake data management system, Step C. Figure 6 shows how the AnIML files are displayed within the Splashlake data management system, with an interactive ^1^H 1D NMR spectra displaying the processed, normalized data collected from the four experimental conditions overlayed. The AnIML file was then used to validate the data, naming conversions, and PPM ranges.

The results from each of the four ^1^H NMR spectroscopy experiments were then summarized in a CSV file, and concatenated into a results summary file, Step D, which contained the following data: PF; MAF; compound 1 identifier; compound 2 identifier (if present); concentration of compound 1; concentration of compound 2 (if present); PRE, experimental repeat number, V type. Steps A-D are from here on referred to as auto factor calculation (AFC).

Finally, details relating to the PF and MAF are added to the summary file by the following criteria. If the MAF is greater than or equal to 1, there is no detectable compound-membrane adhesion event, whereas if the MAF is less than 1, a compound-membrane adhesion event is present. The strength of these interactions increases as you move from 1 to 0. Similarly, if the PF is less than or equal to 1, there is no detectable membrane permeabilization event by the drug (candidate), whereas if the PF is greater than 1, there is evidence that the drug/drug candidate has successfully permeabilized the phospholipid membrane of the vesicle.^17^

### Manual data processing of NMR spectroscopy to calculate PF and MAF values

**Figure 7:**
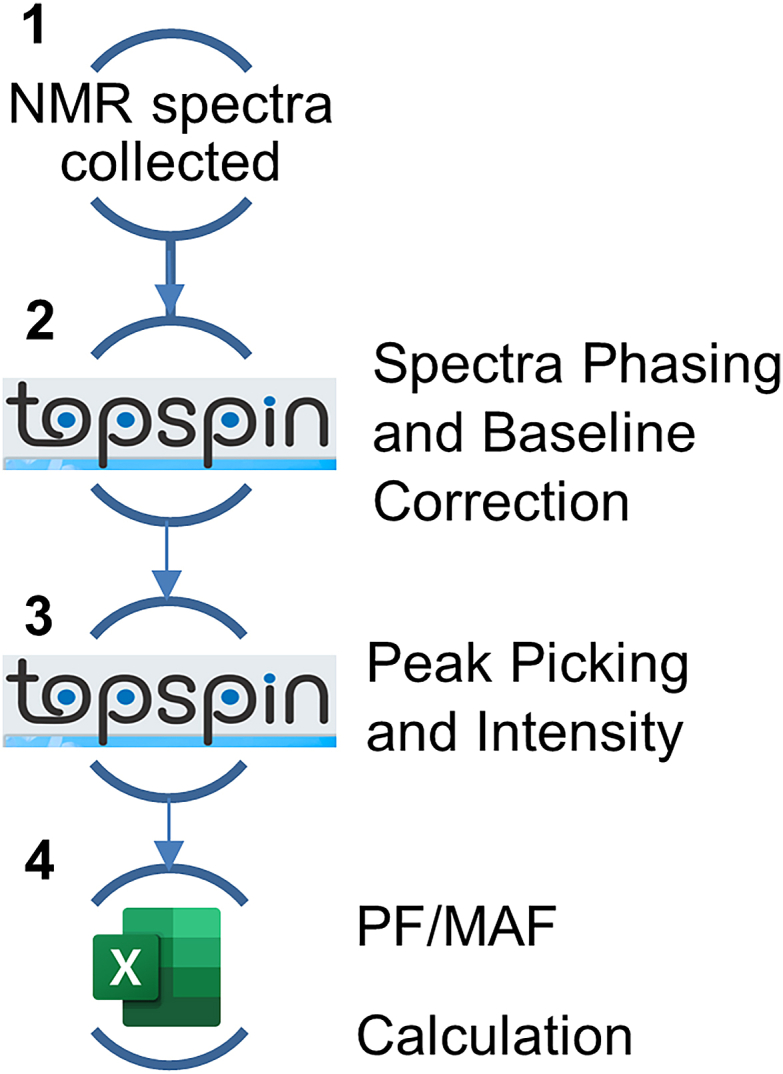
A flowchart overviews the ^1^H NMR spectroscopy assay workflow without automations.

Spectra collected and transformed from a. fid file to a 1D spectra file, Step 1, Figure 7. Spectra undertake auto-phasing (AP). Additional manual phasing (MP) was undertaken as necessary, Step 2, Figure 7. Peaks were picked, and peak intensities were calculated using Topspin, Step 3. The peak intensities were then copied into a spreadsheet template to enable the calculation of MAF and PF values, Step 4. Steps 3–4 are from here on referred to as manual factor calculation (MFC).

### Validation methods

**Figure 8:**
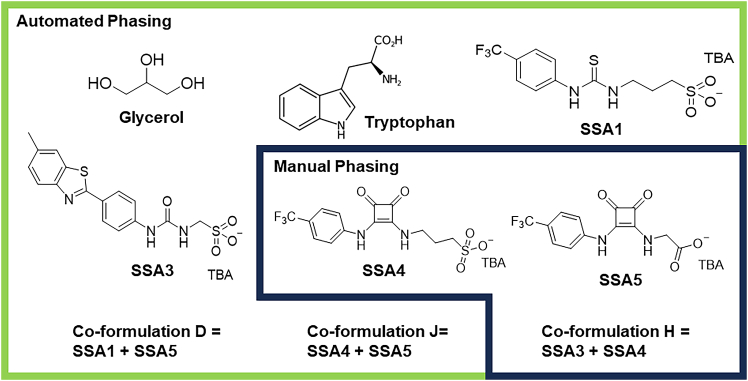
The structure and chemical composition of molecules used within each validation dataset.

To verify the accuracy of the automated and manual processing workflow, results were validated against a subset of accepted, quality-assured published data.^17^^,^^18^ They were also compared to manual processing repeats, completed herein, using a group of researchers using the same spectra used for the generation of published results. The validation dataset comprised nine different experiment examples, including controls, compounds, and co-formulations containing multiple compounds. The structures and compositions of which are shown in Figure 8. Through this process, we are also able to assess the limitations and reproducibility associated with the calculation of MAF and PF values using both manual and automated data processing methods. Each experiment is discrete with unique PPM ranges. As we are not reinterpreting the scientific findings of the original work, the most complex spectra to be MP were selected for incorporation within this study to most challenge the algorithm developed within this work and to better understand the largest impact of phasing on the MAF and PF values.

**Figure 9:**
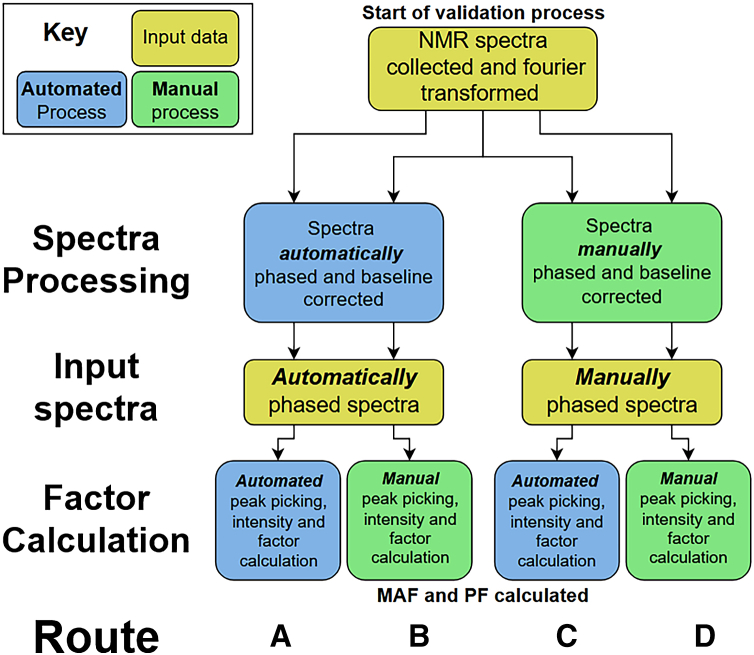
Depicts the four combinations of manual and automated processing used to validate the accuracy of the automated processing workflow and the impact of phasing on the MAF and PF.

There are four possible validation process routes to process the NMR spectra to calculate the MAF and PF factors outlined in Figure 9. Route A: is AP followed by automatic factor calculation (AFC). Route B: is AP followed by MFC. Route C: is MP followed by AFC. Route D: is MP followed by MFC.

These four distinct routes of analysis are used to validate the automated processing workflow. Our aim was to see if the automated processing workflow (Route A and Route C) could replicate results from manually processed validation results (Route B and Route D). Also comparing with results up to publication standard, where the spectra had been correctly phased and therefore assumed to be the most accurate (Route D).

First, we compare the results from MFC (*N* = 8 repeats per experiment), Route B, with the results from AFC, Route A, where the input spectra are both AP, and factor calculation is the only cause of variation. We also visualize these results with published results^18^ (Section: Comparing Route A to Route B to verify MFC and AFC effectiveness).

Next, we explore the variation that phasing has on the MAF and PF. We do this by comparing Route A results to Route D (*N* = 6 repeats per experiment) to explore some instances when the value calculated from Route A was larger than the upper quartile of the validation set for Route D. (Section: Comparing Route D with Route A to assess the impact of phasing).

We wanted to explore whether this was due to factor calculation or differences in the input spectra due to differences between MP and AP. To do this, we took a subset of MP validation spectra (*N* = 3 repeats per experiment), ran these through the AFC algorithm (Route C) with constant PPM ranges. Therefore, the only variation was in the input spectra. We compared Route C with the results from Route A and published results overlaying the signal-to-noise published error. To explore the reasons for the differences found, we explored the results for **co-formulation H** for Route A and Route C, where there is no variation in factor calculation, as both are completed using AFC. Therefore, the only variation is between MP and AP (Section: Exploring Route D and Route C and the co-formulation H experiment).

Finally, we compare the time taken to run the MFC and AFC to get an idea of the time saved from using AFC (Section: Time saving on analysis due to AFC when compared with MFC).

## Results

For conciseness, the results from the manual validation have been summarized in Table 1, we have also included other controls and SSAs which are not part of the validation to allow for a more complete comparison. Generally speaking, AFC has an agreement with MFC within published error, examples where this is not the case are explained herein.

**Table 1:** PF and MAF values calculated through manual factor calculation (MFC) processes and via automatic factor calculation (AFC)

Experimental dataset	MAF MFC	MAF AFC (Route A)	MAF Published (Route D)	PF MFC	PF AFC (Route A)	PF Published (Route D)
**Automatically phased**^**1**^**H NMR spectra (Route B for MFC)**						
**Glycerol**^17^	0.88 ± 0.03	0.88	0.9	1.08 ± 0.03	1.08	1.1
**Tryptophan**^17^	0.94 ± 0.03	0.92	1.0	1.04 ± 0.02	1.06	1.0
**SSA1**^18^	0.94 ± 0.01	0.96	0.94	1.01 ± 0.32	1.04	1.01
**Co-form D**^18^	0.89 ± 0.05	0.79	0.83	1.05 ± 0.11	0.90	1.03
**Co-form J**^18^	0.53 ± 0.17	0.53	0.56	1.13 ± 0.35	1.29	1.11
**Manually phased**^**1**^**H NMR spectra (Route D for MFC)**						
**SSA4**^18^	0.33 ± 0.04	0.39	0.38	1.48 ± 0.82	1.68	1.48 ± 0.3
**SSA5**^18^	0.97 ± 0.53	0.78	0.38	0.71 ± 0.34	0.84	0.67
**Co-form H**^18^	1.44 ± 0.35	1.51	1.66 ± 0.3	0.55 ± 0.36	0.82	0.52
**Spectra processed for comparison with the published values**						
**Ethanol**^17^	Not undertaken	0.98	1.0	Not undertaken	1.44	1,5
**HCT**^17^	Not undertaken	0.69	0.7	Not undertaken	1.32	1.3
**Tryptophan**^17^	Not undertaken	0.92	1.0	Not undertaken	1.06	1.0
**SSA 2**^18^	Not undertaken	0.88	0.88	Not undertaken	0.79	0.85
**SSA 3**^18^	Not undertaken	0.00^a^	0.00	Not undertaken	0.00^a^	0.00

Error quoted represents ±1 SD. This error cannot be calculated for the route A data, as there is no variation given the same input spectra. Published MAF and PF values, obtained through manual processing methods. Values provided for co-formulations (co-form) are representative of the mixture, unless only one molecule is detectable. PPM ranges in the AFC reflect this. Published error unless otherwise stated: PF = ±0.1; MAF = ±0.05.

a SSA 3 adhered to the vesicles to the point that no signal was seen within the spectra.^18^

### Comparing Route A to Route B to verify MFC and AFC effectiveness

**Figure 10:**
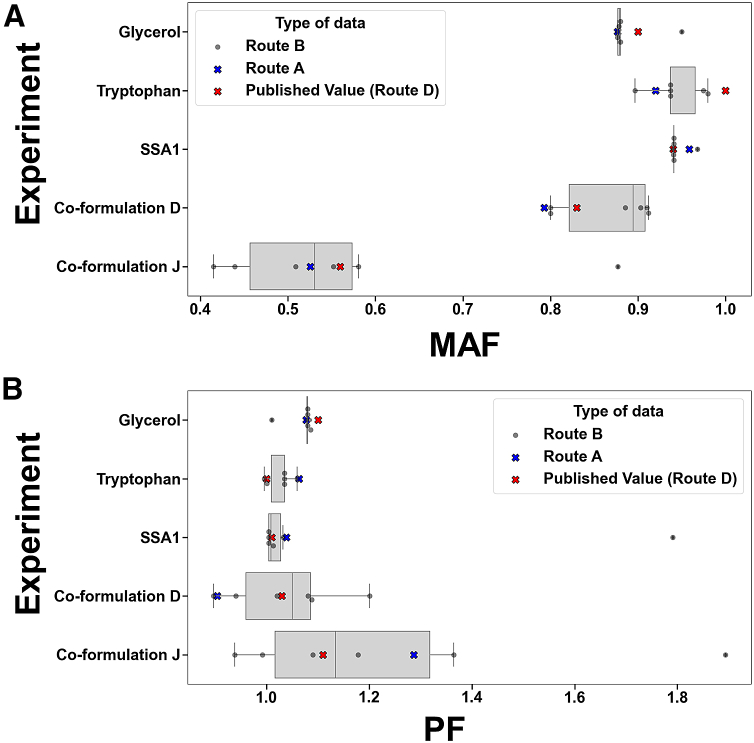
Boxplots of MAF and PF of Route B values produced by 8 different researchers Individual MAF (A) and PF (B) values calculated from Route B by each of these 8 researchers are illustrated by the gray dots. Values produced from Route A (blue, Table 1) and previously published values (red, Table 1) have been superimposed to enable comparison but are not included in boxplots.

A validation dataset of *N* = 8 experimental repeats is used to compare Route B processing with Route A processing. This is used to directly compare AFC and MFC as the input spectra are the same for both. The results are visualized in Figure 10, where both MAF and PF validation datasets are presented individually and summarized as boxplots.

When considering the processing of this workflow, two sources of uncertainty were identified. These sources of uncertainty centered around the manual phasing and “peak picking” analysis of the ^1^H NMR spectra used to calculate the PF and MAF values. This is because these processes are subject to the interpretation and judgment of the individual researchers performing these data processing tasks as to whether they have been completed accurately or not.^22^ When “peak picking,” uncertainty can arise due to the researcher’s discretion relating to the number of NMR resonances to undergo “peak picking” within the PPM range defined for that experiment, which is also defined by the researchers undertaking the MFC and standardized across AFC analysis. The variations in Route B processing were solely due to the MFC, peak picking, intensity calculation, and use of the processing templates to calculate PF and MAF, as the input spectra are consistent.

Figure 10 shows how controls **Glycerol** and **Tryptophan** had the lowest standard deviation (SD), with the more complex co-formulation spectra showing the highest variation, see Table 1. This is to be expected as the controls are fewer complex spectra to process. This displays the assumed knowledge required as well as the variations that can occur within MFC, particularly regarding more complex spectra. We explored the data for some of these outliers and found the variation was due to peak selection. The number of peaks chosen from each range is not consistent across each spectrum within the experiment. This highlights the need for reproducibility, consistency of PPM range selection, and high-quality summaries of each experiment, assured using the AFC workflow. Both published and Route A data are within the spectra signal-to-noise error of the spread of the Route B validation subset (PF = ±0.1; MAF = ±0.05) and within the SD of the median value for all points, aside from PF **SSA1,** which we deem to be within acceptable rounding error, see Table 1.

### Comparing Route D with Route A to assess the impact of phasing

**Figure 11:**
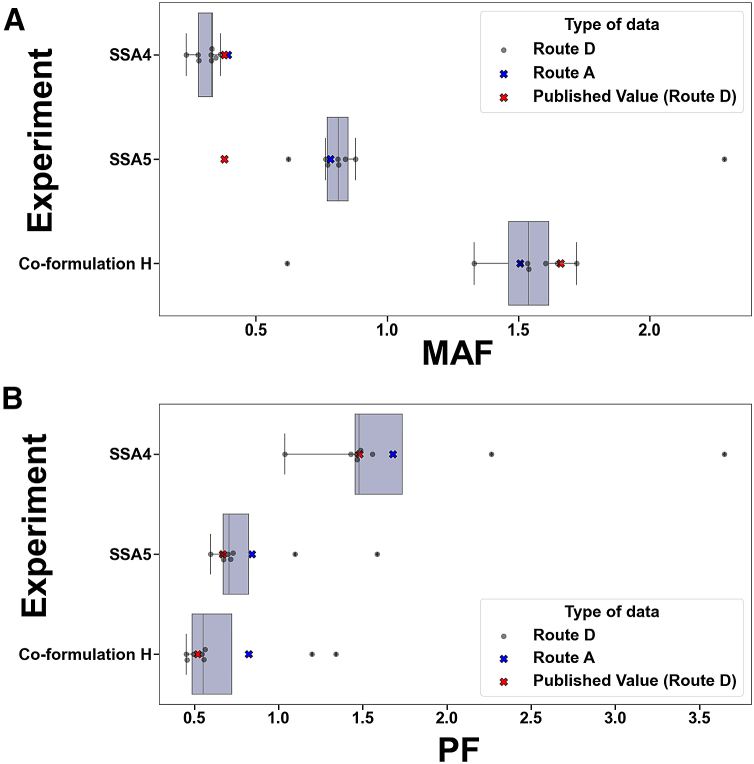
Boxplots of MAF and PF Route D values produced by 6 different researchers Individual MAF (A) and PF (B) values calculated from Route D by each of these 6 researchers are illustrated by the gray dots. Values produced from Route A (blue, Table 1) and previously published values (red, Table 1) have been superimposed to enable comparison but are not included in boxplots.

Having verified that the AFC was producing results within an acceptable spread of the MFC values given the same input spectra, we have compared the Route D to the Route A results as well as published results, as shown in Figure 11.

There is increased SD in the Route D validation dataset compared with Route B, see Table 1, which is to be expected given the added variability of MP. These results can be impossible to reproduce using current MFC methods as the raw analysis templates, spectra files are not published and often lost when the researcher running the analysis moves on. Within the AFC, the PPM ranges, spectra, and results files required for reprocessing are centralized with a robust naming convention, making this less likely.

Errors in calculated MAF and PF due to sample preparation (e.g., pipetting error) also impact the validity of results, in addition to the analysis of the experimental data itself. With further errors produced through the manual data copying/processing and the saving of spectra files (such as may be the case for the MAF values generated originally for **SSA5**). Though never possible to completely remove, manual errors will be greatly reduced in the AFC with a centralized automated processing workflow due to the greater ease for checking results, robust file naming conventions, and sharing of files. The automated generation of a summary file and relevant researcher checks help ensure correctness.

When exploring the MFC templates, we found that some of the outliers (MAF and PF values outside the whiskers of the boxplots) in Figure 11 were due to intensity measurements not reducing by a constant amount, likely due to phasing and baseline correction causing variation in the MAF and PF, as well as inconsistencies in the number of peaks selected across the experiment, as mentioned above.

### Exploring Route D and Route C and the co-formulation H experiment

**Figure 12:**
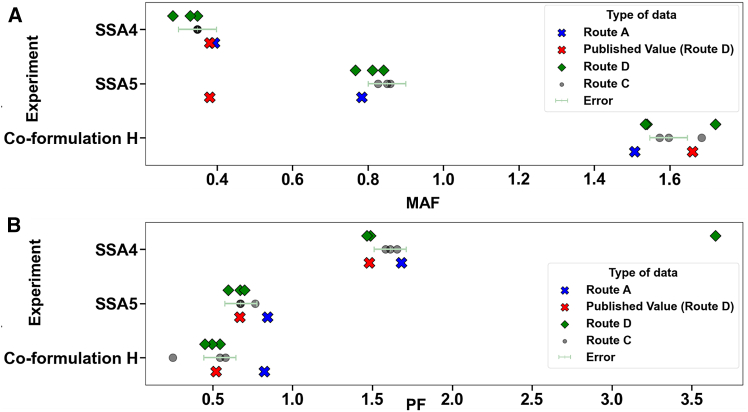
MAF and PF values calculated from Route C using manually phased spectra from 3 separate researchers as input Values produced from Route A (blue, Table 1) and previously published values (red, Table 1) the signal to noise error (PF = ± 0.1; MAF = ± 0.05) has been superimposed over the median value of the Route C subset. Finally, we have displayed the same spectra analyzed by MFC Route D spectra to contrast any differences.

Route A overpredicts PF when compared to Route D processing, as shown in Figure 11. To understand whether the AFC algorithm or the input spectra caused these inaccuracies in PF, we compared the results from Route C processing. Figure 12 shows the subset of *N* = 3 experiment analysis repeats for **SSA4**, **SSA5** and **Co-form H**. The ^1^H NMR spectra were phased by three separate researchers with a constant PPM range used by the algorithm, and the input spectra were the only cause of variability. Route D results from the same input spectra as Route C, the error in the signal-to-noise ratio centered on the median Route C value, published values, and Route A values were also added for comparison.

Promisingly, Figure 12 shows, for all Route C calculated PF and MAF values, aside from Co-form H, all repeats fall within the signal-to-noise error of the spectra, meaning the AFC algorithm is working as intended for this subset. Generally showing less variation the Route D subset due to reduced uncertainty caused by MFC and no longer skew toward over estimating PF values (as it did with Route A), and it can therefore be concluded that these variations were due to the input spectra and not the AFC algorithm. Another example demonstrating the added consistency that comes with the AFC algorithm is the PF outlier associated with **SSA4,** which is solely due to MFC.

**Figure 13:**
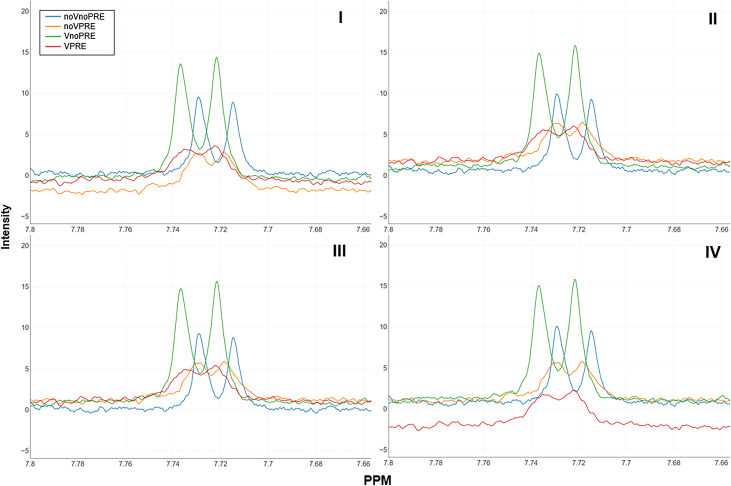
^1^H 1D NMR spectra displayed within the PPM range, 7.7–7.8, associated with co-formulation H to examine cause of error in the MAF and PF values cause by baseline correction error during phasing: Route A (I) giving the overestimate in PF, (II) and (III) Route C giving the expected PF value, (IV) Route C giving the underestimate in PF for **Co-formulation H** in Figure 12

We explored the results of **Co-form H** to understand why the PF is calculated lower or higher than the expected error. To do this have taken the spectra as displayed on the AnIML summary files for the Route A and Route C processing of the spectra. We have displayed the PPM ranges passed to the AFC in Figure 13. Where Figure 13I displays the AP Route A input spectra, Figure 13II, III, and 13IV show the variation in input processed spectra due to manual phasing.

Figure 13I shows why the PF was larger than the signal-to-noise error for Route A processing. The noVPRE peak intensities are lower when compared to the Route C repeats, due to this spectrum being out of phase. Figure 13IV, in contrast, has phasing error due to manual spectra processing in the VPRE 1H 1D NMR spectra (red), causing this intensity to be significantly lower for PF calculation and, in turn, the PF value to be smaller than the signal-to-noise error. Where the ^1^H 1D NMR spectra are correctly MP by the researcher are used as input, as seen in Figures 13 II and 13III, the automated 2processing workflow provides two results that fall within signal-to-noise error. The results shown in Figure 13 are shown in Table 2.

**Table 2:** Shows the manual and automated processing results for the subset of manually phased experiments

	Auto (Route C)			Manual (Route D)		
	**MAF**					
	**SSA4**	**SSA5**	**Co-formulation H**	**SSA4**	**SSA5**	**Co-formulation H**
**Published**	N/A	N/A	N/A	0.38	0.38	1.66
**I (Route A)**	0.39	0.78	1.51	N/A	N/A	N/A
**II**	0.35	0.85	1.60	0.35	0.84	1.54
**III**	0.35	0.83	1.69	0.33	0.81	1.72
**IV**	0.35	0.86	1.57	0.28	0.77	1.54
	**PF**					
**Published**	N/A	N/A	N/A	1.48	0.67	0.52
**I (Route A)**	1.68	0.84	0.82	N/A	N/A	N/A
**II**	1.58	0.67	0.58	3.37	0.60	0.55
**III**	1.66	0.67	0.54	1.49	0.67	0.50
**IV**	1.61	0.77	0.25	1.46	0.70	0.45

Spectra shown in Figure 13.

This phenomenon is more prevalent in PF calculation as the factor is calculated using two spectra that contain PREs, which broadens the peaks, making them less intense and hard to separate, thereby increasing the noise. This also reduces the intensity of the peaks, causing any error in phasing or baseline correction to be more prevalent. Due to the PF and MAF value being displayed below the spectra in the AnIML xml files, these spectra can be readily downloaded and reprocessed at any time due to the data being centralized.

### Time-saving on analysis due to AFC when compared with MFC

To verify the time saved using the AFC processing, we also recorded the time taken for both MP, MFC, and AFC. The time taken for the AFC process was recorded twice: the total process (first run of analysis), and AFC only (rerunning of analysis). Total process is the time taken for file ingest, AnIML conversion, processing, and automated upload of results back to Splashlake and reprocessing, automated upload of results back to Splashlake.

To obtain the time taken for the MP of all four ^1^H NMR spectra included with an experimental dataset, the time at which the spectral files were opened in Topspin was recorded at T = 0. The spectra were then MP by the researchers (*N* = 6 repeats per) to a standard deemed acceptable to the criteria provided by the original research team members that designed the assay. At this point, the timer was stopped.

**Figure 14:**
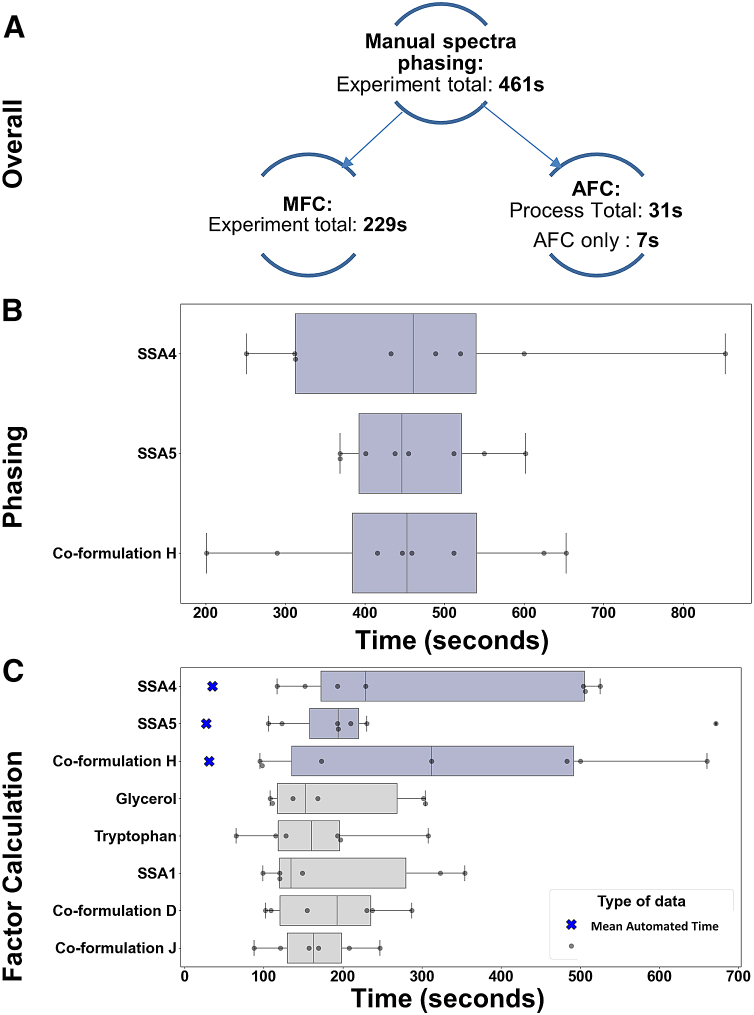
The average time taken to process each section of the workflow (A) Overall: A flowchart to show the time taken for each section of the workflow. (B) Phasing: A boxplot to show the time taken for the phasing of the 1H 1D NMR spectra. Individual values for the time taken to phase are shown within the boxplot. (C) Factor Calculation: A boxplot to show the time taken for the peak picking and MAF and PF calculation of the 1H 1D NMR spectra. Light-blue gray shows time for MP, with gray spectra having only undergone AP. The mean time for the AFC, including file upload and basket creation, is shown as a blue cross.

The time taken for MFC, T = 0, began with all four ^1^H NMR spectra for an experiment loaded in Topspin, where peaks are picked and intensity calculated. The values are copied into the factor calculation template spreadsheet. Once the MAF and PF values are calculated, the timer was stopped. The time taken for these processes is overviewed in Figure 14.

Overall, the mean time saved is 223s on factor calculation between the two methods just to run the processing. With a minimum time save of 57 s and a maximum time save of 657 s. Variations are due to the complexity of the analysis and the researcher's NMR experience. These results are also shown in Table 3. The time taken to curate and load files was not accounted for within our manual processing timings. This includes time taken to copy, curate, and load the data, or that the PPM ranges were already given in a premade template from published work. Therefore, the time saved from using the automated processing workflow is likely greater than the results show, as these processes are encompassed within the automated workflow. Though this would increase the time taken for AFC from 6 s to 31 s, the time to complete this extra process manually would be much greater.

**Table 3:** Shows the mean, median, maximum and minimum time taken for MP, MFC, and AFC

Process	Mean time/s	Median time/s	Max time/s	Min time/s
**Manual phasing**	461	451	853	201
**Manual factor calculation**	229	171	660	65
**Automated factor calculation reprocessing**	6	7	8	3
**Automated factor calculation processing**	31	28	46	25

### Other benefits of the AFC workflow

Along with the time-saving benefits, automation of the workflow has other advantages when it comes to data quality. To create the automated workflow, we implemented naming conventions that make the data searchable and findable if reanalysis is needed later. The time required to curate the subset of data for validation was in the time frame of months, which would not be the case if the data had been processed using the automated workflow within a data management system. For the manual processing, the PPM ranges are only recorded within the Excel template and are not easily accessible, whereas in the automated workflow, this data is available and editable within the data management system, allowing the analysis to be rerun quickly and easily.

**Figure 15:**
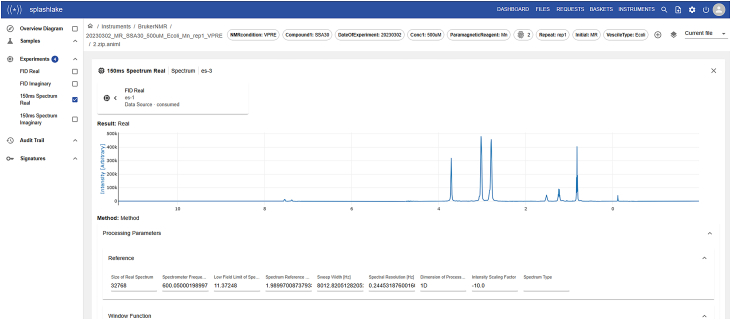
Shows an AnIML file containing raw spectra data and metadata is displayed within Splashlake Hub The NMR data is displayed as an interactive spectrum along with metadata from the spectra file. Automatically extracted metadata from the filename is shown across the top. The different types of spectra, FID, transformed and normalized as options to be displayed down the side.

Within the AFC workflow, the correctly phased ^1^H 1D NMR spectra files are available for download for reuse and are viewable in AnIML XML file representations, such as that exemplified in Figure 15. The raw spectra along with experiment summary files, which are automatically saved and searchable within the data management system, are used to generate machine accessible data for cross-project analysis with other assays on the same project.

Human error is impossible to eliminate, though we have aimed to reduce it within the workflow. The reduced time for analysis makes results easier to rerun with summary reports containing spectra generated. Generation of summary files and automated spectra upload reduces errors occurring due to copying data. Phasing of spectra is easily verified using the AnIML file experiment summarizes.

This process also allowed us to refine the factor calculation methodology of the assay and showed the importance of considering and recording these methods for developing assays so they are repeatable without assumed knowledge of the researcher processing the data. For example, during the manual validation, the TopSpin command line phasing was refined to the method stated within this paper and will be used for results going forward. The algorithm also has the ability to integrate and peak pick through magnitude or absolute peak intensity. The PPM ranges can be adjusted to measure each multiplet as a whole or all peaks within a multiplet. These can be more easily explored with the more controlled AFC.

### Findings

We have automated and validated a complex ^1^H NMR spectroscopy-based assay used to determine the adhesion of chemical compounds (drugs/drug candidates) to (MAF) and permeation (PF) through a phospholipid vesicle membrane. Through the incorporation of automated processes within the data analysis workflow, we have increased and defined the reproducibility of this assay while simultaneously providing an audit trail for drug candidate data. Allowing for the MAF and PF to be reprocessed if necessary. This is achieved by making the raw ^1^H NMR spectra and accessible alongside experiment summary files centralized and searchable within a data management system.

To support the automation of this workflow, we present an example naming convention required to implement the calculation of MAF and PF values from the raw NMR data within a lab environment. We show that through outputting high-quality, machine accessible data, we increase the ease of human and computational analysis, as well as reducing the implied knowledge required to carry out the analysis.

We have validated both the AFC and previously published results, and calculated results within the manual validation subset. In turn, we have increased understanding of the causes of error within the assay, and refined and built in procedures to reduce these errors in the automated processing, showing the benefit of automating these processes in refining scientific processing methodologies.

Through our validation processes, we have shown that automating the processing of these data results in an average time saving in analysis of 223 s per experiment, with variation dependent on research experience and complexity of ^1^H NMR spectra. This automated, centralized and searchable approach to processing and storing the data associated with this assay lays the groundwork to enable the adoption of this assay into high-throughput drug discovery pipelines, to support drug development, decreasing the costs and time associated with the development of a clinical candidate molecule(s).

### Limitations of the study

Time recorded for the processing workflows did not include time to capture, rename, move, and copy data files, meaning the time taken will be longer than recorded. The AFC is heavily dependent on the quality of the initial spectra processing ingested into the workflow. This is demonstrated in the co-formulation H example, where errors in AP or baseline correction led to errors in PF and MAF values. Finally, the workflow is reliant on the researcher specifying the PPM ranges correctly for each compound.

## Resource availability

### Lead contact

Further information and requests for resources and reagents should be directed to and will be fulfilled by the lead contact, Thomas Allam (ta1u18@soton.ac.uk).

### Materials availability

This study did not generate new spectra or reagents.

All validation datasets used for the automated NMR workflow were sourced from previously published work by Serrano-Sanchez et al.^17^ and Hilton et al.^18^

### Data and code availability

• All original code and data have been deposited at - Allam, T. (2026). Data and scripts associated with “Automation and validation of an NMR spectroscopy-based drug discovery assay.” University of Southampton. Dataset. https://doi.org/10.5258/SOTON/D3943 - and is publicly available as of the date of publication.

## Acknowledgments

T.L.A. would like to thank the University of Southampton and UKHSA Grant-in-aid funding for the UKHSA Open Innovation Platform (Project 113361). J.F. and S.P.K. would like to thank P.S.D.I. L.J.W., K.L.F.H., and J.R.H. would like to thank the UKRI for a Future Leaders Fellowship awarded to J.R.H. (MR/T020415/1, MR/Y03385X/1). O.B.K., M.S., and M.R. would like to thank the SoCoBio DTP (BB/T008768/1) for funding. P.P. and D.B. would like to thank the University of Kent for funding. P.N. would like to thank the Nelson-Mandella, Leverhulme funded PhD studentship. Would like to thank Prof. Mark Sutton and Dr Charlotte Hind (UKHSA, UK) for researcher training and support. The authors acknowledge Splashlake for help with data infrastructure.

## Author contributions

Conceptualization, T.L.A., J.R.H., J.F., and S.P.K.; methodology, T.L.A., J.R.H., J.F., M.R., J.O.R., and S.P.K.; investigation, T.L.A., J.R.H., J.F., M.R., and J.O.R.; writing – original draft T.L.A., J.R.H., J.O.R., and S.P.K.; manual validation, T.L.A., L.J.W., K.L.F.H., P.P., P.N., D.B., M.S., O.B.K., M.R., and J.R.H. writing – review and editing, T.L.A., L.J.W., K.L.F.H., P.P., P.N., D.B., M.S., O.B.K., M.R., J.O.R., and J.R.H.; funding acquisition, J.F., C.H., and M.S.; supervision, J.R.H., J.F., C.H., M.S., P.H., and S.P.K.

## Declaration of interests

The authors declare no competing interests.

## STAR★Methods

### Key resources table

**Table:** 

REAGENT or RESOURCE	SOURCE	IDENTIFIERS
**Deposited Data**		

Previously published validation data	Serrano-Sanchez et al.^17^ 2024	https://doi.org/10.1039/D4CC01515K
Previously published validation data	Hilton et al.^18^ 2025	https://doi.org/10.1039/D5TB00653H
Python code and data	Allam, T. (2026). Data and scripts associated with ‘Automation and validation of an NMR spectroscopy-based drug discovery assay'. University of Southampton. Dataset and code.	https://doi.org/10.5258/SOTON/D3943

**Software and algorithms**		

Python 3	Python Software Foundation	https://www.python.org; RRID: SCR_008394
Topspin	Bruker	RRID:SCR_014227

### Method details

The AFC was executed by the Python workflow, supporting DMS framework, validation method and validation dataset used, summarised herein, is explained in detail in the STAR Methods section of the paper.

All ^1^H NMR spectra were acquired using a Bruker 600 MHz spectrometer. For each drug candidate or co-formulation, a complete experiment consisted of four distinct conditions varying by the presence of vesicles, paramagnetic relaxation enhancement agents, and the compound. Raw data were collected as.fid files.

Primary spectral processing was performed using TopSpin software^20^ using the following sequence of commands: Fourier Transformation, efp (Exponential multiplication, Fourier transform and apply phase corrections). Phase Correction, apk (Automatic phase correction of the spectrum). Baseline Correction, abs (Automatic baseline correction and integration of major signals in spectrum).

The workflow utilised Splashlake Software as the DMS. Data ingest was triggered by a monitored folder system. Metadata extraction was completed from a standardised file naming convention using regular expressions to automatically tag files with compound identifiers, concentrations, and repeat numbers. 1D spectra files were programmatically converted into AnIML format to ensure long-term data accessibility and support interactive visualisation within Splashlake.

To ensure reproducibility and experimental validity, the automated workflow was benchmarked using a subset of experiments on 9 compounds and coformulations from quality-assured data from published work from Serrano-Sanchez et al.^17^ and Hilton et al.^18^ Though not available through the original work, the raw spectra were obtained and available with the code for this paper (see key resources table).

Scientific findings from original studies were not reinterpreted; instead, the corresponding raw ^1^H NMR spectra were reprocessed to ensure the automated results fell within the previously published signal to noise error margins (PF = ±0.1; MAF = ±0.05) as well as the spread of experimental Route B repeats. Outliers identified during the validation process were analyzed to understand their causes as outlined in the section: Exploring Route D and Route C and the Co-formulation H experiments.

### Quantification and statistical analysis

The variation in manual factor calculations and manual phasing were repeated by multiple independent researchers. The results were summarised and processed using Python to generate boxplot comparison figures, means and standard deviations for the manual validation datasets. Boxplots were selected to visualise the data because they visualise outliers as being outside the whiskers of the boxplots, according to the formulas below:

$${Lower\;outlier<Q}_{1}-1.5\cdot \text{IQR}\;and\;{Upper\;outlier\;>Q}_{3}+1.5\cdot \text{IQR}$$

The influence on the boxplots from the outliers are visible in the quartile ranges and allow the display of the entire validation datasets and major outliers without needing to omit them from the dataset.

The repetitions were used to establish variation in manual processing. Route B (AP + MFC), *N* = 8 experimental repeats per condition to assess the variation of MFC and validate that Route A results fall within this variation. The results for MFC validation are displayed as MEAN ±1 SD in Table 1.

Route D (MP + MFC), *N* = 6 repeats per condition to establish the variation due to MP and MFC. The results for MFC validation are displayed as MEAN ±1 SD in Table 1.

Route C (MP + AFC), *N* = 3 repeats to understand the impact of phasing on the algorithm. The individual results are described in Table 2.

The time taken for AFC, MFC and MP has the mean, medium, maximum and minimum outlined in Table 3.
